# Low-temperature precipitation synthesis of flower-like ZnO with lignin amine and its optical properties

**DOI:** 10.1186/1556-276X-8-431

**Published:** 2013-10-17

**Authors:** Ting-Ting Miao, Dong-Xiao Sun, Yuan-Ru Guo, Chuan Li, Yan-Li Ma, Gui-Zhen Fang, Qing-Jiang Pan

**Affiliations:** 1 Key Laboratory of Bio-based Material Science & Technology (Ministry of Education), Materials Science and Engineering College, Northeast Forestry University, Harbin 150040, China; 2 Key Laboratory of Functional Inorganic Material Chemistry (Ministry of Education), School of Chemistry and Materials Science, Heilongjiang University, Harbin 150080, China

**Keywords:** One-step synthesis, Lignin amine, ZnO nanocrystallites, Photoluminescence

## Abstract

A facile precipitation method has been developed to synthesize ZnO with [bis(2-aminoethyl)amino]methyl lignin (lignin amine) that is chemically modified from low-cost pulp industrial lignin. The obtained ZnO crystallites have been characterized to exhibit a hexagonal wurtzite structure, and their sizes have been determined at *ca*. 24 nm (mean value). These ZnO nanocrystallites are of high purity and well crystallized. Our present synthetic approach apparently exempts the commonly used calcining purification procedure. It is found that the morphology of ZnO and its specific surface area are capable of being tuned by varying the added lignin amine amount. Using the optimal 10 mL lignin amine, the synthesized ZnO exhibits flower-like morphology with proper specific surface area. Additionally, photoluminescence property of the obtainable ZnO displays two emissive bands at 383 nm (sharp) and in the range of 480 to 600 nm (broad) at room temperature. Their intensities were revealed to depend on the added lignin amine amount as well as on the molar ratio of Zn^2+^/OH^-^. The present investigation demonstrates that our method is simple, eco-friendly, and cost-effective for the synthesis of small-size ZnO materials.

## Background

Zinc oxide is an important n-type semiconductor with a wide band gap of 3.37 eV, allowing for its wide applications in optoelectronic and microelectronic devices [1-3]. Due to its small size and large specific surface area, nanoscale ZnO has showed superior performance relative to the bulk one [4,5]. Up to date, improving the synthetic methods of ZnO nanomaterials as well as developing new ones has been increasingly attractive. These methods do affect the properties of materials such as field emission, optics, piezoelectricity, and catalysis [6-8]. Although ZnO materials with different morphologies were synthesized [1,9-14], some disadvantages still remain in the present methods. Therefore, a facile, cost-effective, as well as environment-friendly approach is highly demanded, especially the approach that produces ZnO in a large scale.

In this work, the controllable synthesis of ZnO nanocrystallites has been developed using a facile precipitation method by tuning the amount of [bis(2-aminoethyl)amino]methyl lignin (i.e., lignin amine). The pure flower-like ZnO nanocrystallites with proper size have been fabricated and fully characterized. Since the used lignin amine is low cost and derives from chemical modification of lignin which is a by-product of pulp industry, a potentially commercial prospect on producing ZnO nanocrystallites is expected.

## Methods

The alkaline lignin (industrial purity) was supplied by Qianjin Fuli Limited Company in Jilin province of China. Formaldehyde and diethylenetriamine (analytical purity) were purchased from Tianjin Kemiou Chemical Reagent Co., Ltd. (Tianjin City, China). Zinc acetate (Zn(CH_3_COO)_2_·2H_2_O, 99% purity) and sodium hydroxide (NaOH, flakes, 97% purity) were used as zinc source and precipitant, respectively. All the chemicals were used as received without any purification.

In our experiment, lignin amine (0.3 g mL^-1^) was synthesized using a previously reported method [15]. Alkaline lignin of 20.0 g was mixed with 40 mL deionized water and 60 g formaldehyde (40% by weight). Diethylenetriamine (40 mL) was added into the stirring mixture solution dropwise at room temperature. After this, the mixture was stirred at 75°C for 3 h by reflux. Then the lignin amine solution with a concentration of 0.3 g mL^-1^ was obtained.

Zinc acetate of 2.7 g was dissolved in 25 mL deionized water. Then a 25-mL NaOH solution (0.08 g mL^-1^) and lignin amine were added to the zinc acetate solution. The mixture solution was stirred for 5 h in the 80°C water bath. Then the mixture solution was cooled to room temperature, and the ZnO particles were precipitated. The precipitate was filtered and washed with deionized water, and the ZnO particles were obtained after drying at 30°C for 12 h. To study the effect of lignin amine on the morphology of the as-prepared ZnO, experiments using 0, 5, 10, and 15 mL lignin amine were carried out. The products were labeled as ZnO-0, ZnO-5, ZnO-10, and ZnO-15. Additionally, ZnO-10 has been chosen to examine whether the commonly used calcining purification procedure is necessary for our synthesized materials. We calcined ZnO-10 in air with a heating rate of 10°C min^-1^ and allowed it to stand at 500°C for 2 h using a compact muffle furnace (KSL-1700X, MTI Corporation, Richmond, CA, USA).

The crystallinity and purity of the prepared samples were analyzed by X-ray diffraction (XRD; Rigaku D/Max-RC, Tokyo, Japan) using CuKα radiation. Scans were performed from 5° to 80° (2*θ*) at a rate of 4° min^-1^. Scanning electron microscopy (SEM) images were taken with a field emission microscope (S-4800, Hitachi, Ltd., Chiyoda-ku, Japan). Transmission electron microscopy (TEM) imaging and high-resolution TEM (HRTEM) imaging of the samples were performed on a JEM-2100 electron microscope (JEOL, Tokyo, Japan) with an acceleration voltage of 200 kV. Carbon-coated copper grids were used as the sample holders. Brunauer-Emmett-Teller (BET) nitrogen adsorption-desorption experiments were carried out on the automated surface area and pore size analyzer. The photoluminescence performance was examined using a fluorescence spectrophotometer (FLS920, Edinburgh Instruments Ltd., Royston, UK) with a Xe lamp at room temperature at an excitation wavelength of 325 nm.

## Results and discussion

**Figure 1:**
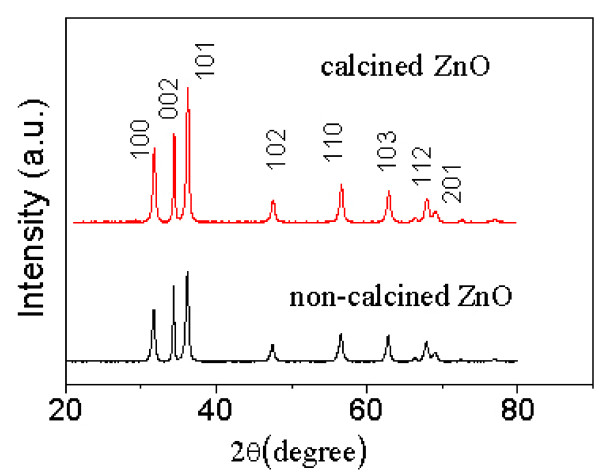
**XRD patterns of calcined and non**-**calcined ZnO-10.**

The typical XRD pattern of ZnO-10 prepared from precipitation (i.e., non-calcined ZnO-10) is presented in Figure 1, compared with that of the calcined ZnO-10. It is shown that the entire diffraction peaks of the non-calcined ZnO-10 match well with those of wurtzite hexagonal phase ZnO (JCPDS card no. 36–1451). Moreover, no peaks of impurities are found. This demonstrates that our ZnO is pure and well crystallized. The intensity of the (002) diffraction is higher than that of (100), which suggests that the as-prepared ZnO has weak preferential growth along the *c*-axis. Meanwhile, both the calcined and non-calcined ZnOs have approximately identical XRD patterns. Their average crystallite sizes were calculated to be about 24 nm using the Debye-Scherrer formula, building on peaks of (101), (002), and (100) planes. Therefore, the further calcining purification usually used in many syntheses is not required anymore while applying our synthetic approach. Moreover, lignin is the second most abundant natural raw material and conveniently available as a by-product of the pulp industry [16]. The method using a lignin derivative to prepare ZnO nanocrystallites is cost-effective.

**Figure 2:**
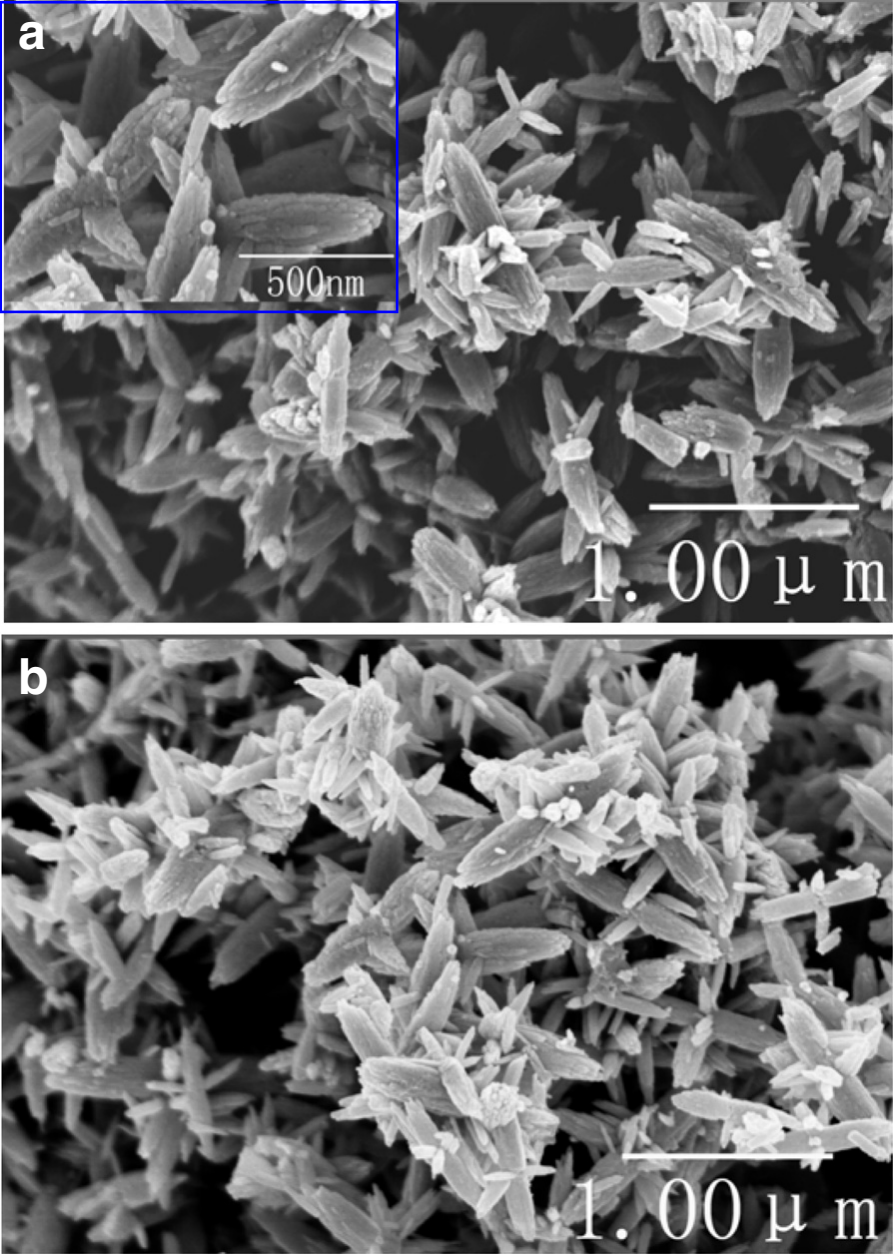
**SEM images of the synthesized ZnO**-**10 nanocrystallites. (a)** Non-calcined; inset, high-magnification image. **(b)** Calcined.

The morphologies of the ZnO nanocrystallites have been analyzed by SEM. Both the non-calcined (Figure 2a) and calcined (Figure 2b) ZnO-10 display the same flower-like nanostructures. This confirms that the calcining procedure is not necessary in the present synthetic route, in agreement with the above XRD results. Therefore, only the non-calcined ZnO samples will be discussed below. From the SEM image (the inset of Figure 2a), ZnO nanocrystallites are observed to exhibit a substructure of several tapered petals. The petal has been determined to be about 200 nm wide and 500 nm long. Many needles grow in each petal from the center of the flower, producing a scabrous surface.

**Figure 3:**
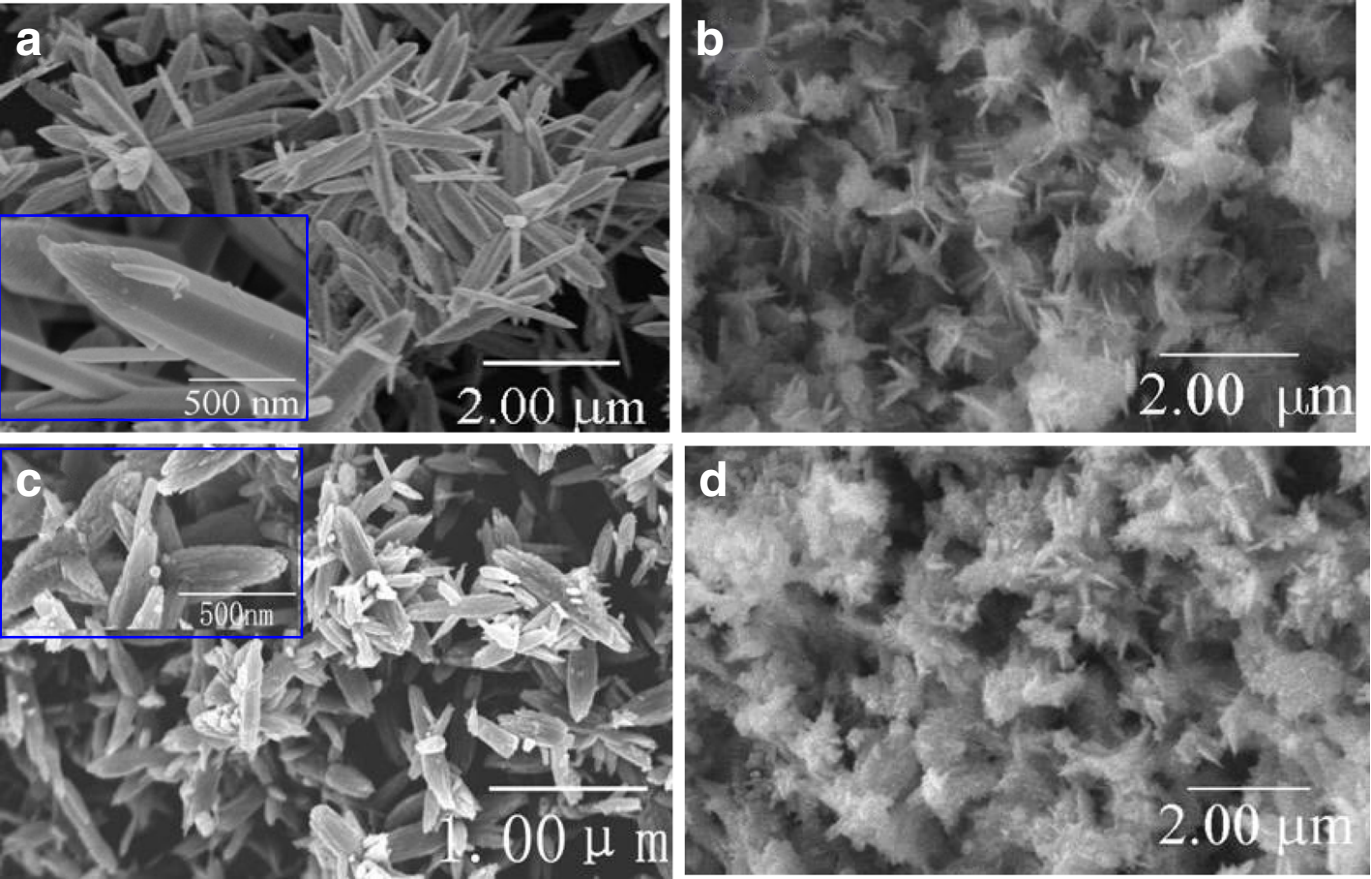
**SEM images of the ZnO nanocrystallites. (a)** ZnO-0, **(b)** ZnO-5, **(c)** ZnO-10, and **(d)** ZnO-15. The insets in **(a)** and **(c)** are high-magnification images.

In order to explore the effects of the added lignin amine on the morphology and specific surface area of the ZnO nanocrystallites, we have synthesized ZnO using 0, 5, 10, and 15 mL lignin amine. The obtained SEM images are given in Figure 3. One can see that the ZnO prepared with 10 mL lignin amine has the most favorable morphology in Figure 3c, although others also exhibit general flower-like structures. ZnO-0 is composed of non-uniform microneedles (Figure 3a), giving rise to the average length of 1.94 μm and very small BET specific surface area of 1.7 m^2^ g^-1^. When using 5 mL lignin amine (i.e., ZnO-5), flowers formed by slices have been observed with not large BET specific surface area of 12.3 m^2^ g^-1^. Regarding the synthesized ZnO-10, both good flower-like morphology (Figure 3c) and a high BET surface area of 21.5 m^2^ g^-1^ have been achieved. When increasing the lignin amine amount to 15 mL, nanosheet structures of ZnO-15 (Figure 3d) were obtained with a surface area of 6.1 m^2^ g^-1^. Therefore, the amount of lignin amine plays a predominant role in determining the morphologies of ZnO nanomaterials as well as controlling their specific surface area.

**Figure 4:**
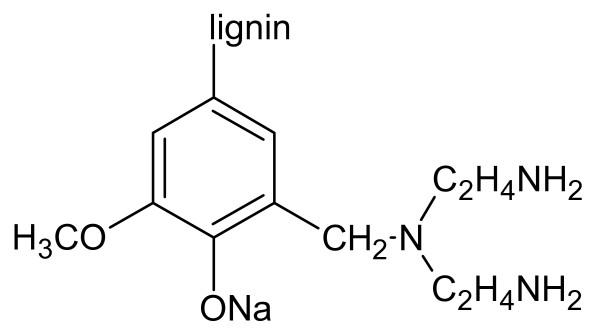
Scheme of [bis(2-aminoethyl)amino]methyl lignin (lignin amine).

It has been well established that the reaction of Zn(CH_3_COO)_2_·2H_2_O with NaOH gives rise to Zn(OH)_2_. A hydrolysis process allows the formation of [Zn(OH)_4_]^2-^, and then the subsequent dehydration yields ZnO [17]. Lignin is a polyphenolic material, arising from the copolymerization of three phenylpropanoid monomers, i.e., coniferyl, sinapyl, and *p*-coumaryl alcohol. Lignin behaves as a macromolecular surfactant and generates capsules in the solution because it is full of hydrophilic and hydrophobic sites. Modification into lignin amine (Figure 4) by diethylenetriamine according to the Mannich reaction greatly improves its surface activity because of introducing hydrophilic amine groups. As a polar crystallite, ZnO is well known to possess partially positively charged Zn^2+^-terminated (001) and negatively charged O^2-^-terminated ($00\overset{-}{1}$) polar surfaces. In the reaction process, the positive face of ZnO is absorbed on lignin amine via the electrostatic attractive interaction, which inhibits the growth of ZnO and facilitates the formation of nanoneedles [18]. If more lignin amine, for instance 15 mL, is added, the inhibition effect is enhanced and eventually results in the formation of ZnO slices. From the above analysis, one can see that the morphology of our ZnO strongly depends on the amount of lignin amine. This allows for the preparation of size-controllable ZnO nanomaterials. In brief, the use of lignin amine and its added amount are indispensable to form small-size flower-like ZnO crystallites.

**Figure 5:**
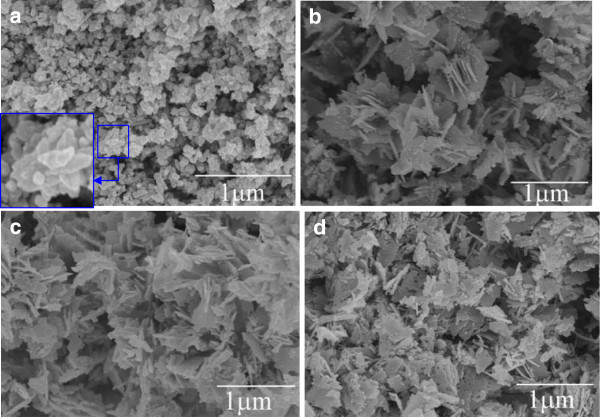
**SEM images of ZnO-10 nanocrystallites at various molar ratios of Zn**^**2+**^**/OH**^**-**^**. (a)** 1:2, **(b)** 1:6 **(c)** 1:8, and **(d)** 1:10. The inset in **(a)** is a high-magnification image.

As the base concentration can affect the morphology of ZnO crystallites, the SEM images of the ZnO prepared with 10 mL lignin amine at various molar ratios of Zn^2+^/OH^-^ were obtained and are shown in Figure 5. As shown in Figure 5a, nanoparticles with diameters of 50 to 100 nm were determined when Zn^2+^/OH^-^ is 1:2. The obtainable flowers do not grow well. This implies that there is no obvious preferential growth direction of ZnO in this condition. When the molar ratio of Zn^2+^/OH^-^ is 1:4, the preferential growth along the *c*-axis direction forms multi-needle flowers, which agrees well with the above XRD results. Continuously increasing the base concentration (Zn^2+^/OH^-^ of 1:6, 1:8, to 1:10, Figure 5b,c,d), all the prepared ZnO nanomaterials feature the morphology of the slices. That is because in the condition of high base concentration, the upper side of the needle-like ZnO will be slowly dissolved during the growth, eventually forming ZnO slices.

**Figure 6:**
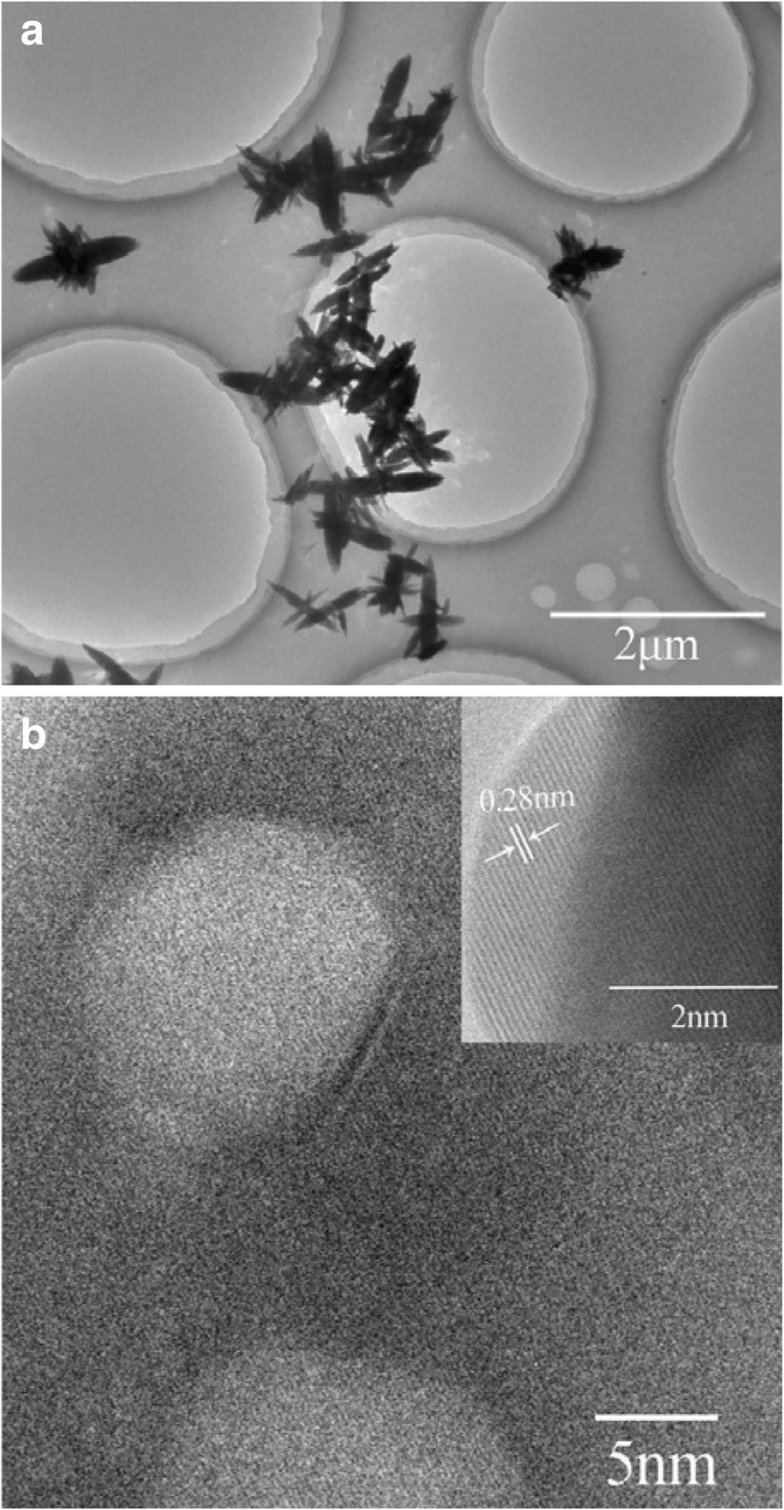
TEM (a) and HRTEM (b) images of ZnO-10.

The detailed structural feature of ZnO-10 has been examined by TEM. Dispersive ZnO nanocrystallites were observed in Figure 6a. Two bigger petals form the fusiform macro-axis, and several minor petals grow perpendicular to the macro-axis, which agree well with the SEM results. Hexagon mesopores with a diameter of 10 nm have been clearly found in Figure 6b, which may affect the properties of the ZnO nanoflowers. Furthermore, the lattice spacing was measured to be 0.28 nm (the inset of Figure 6b), attributed to the interplanar spacing of the wurtzite ZnO (100) plane.

**Figure 7:**
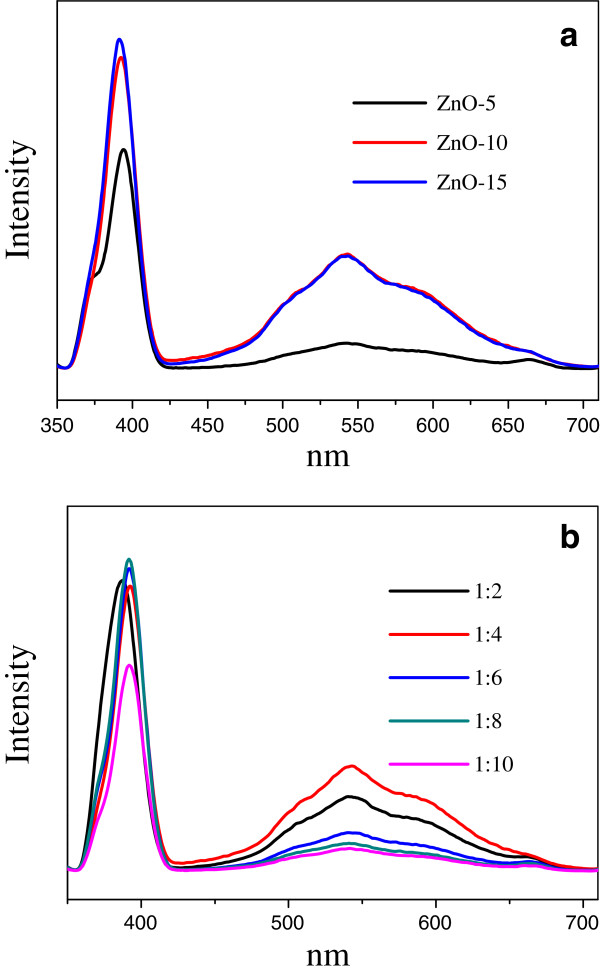
**PL spectra of the prepared ZnO. (a)** By various lignin amine amounts at 1:4 of Zn^2+^/OH^-^ and **(b)** by various Zn^2+^/OH^-^ ratios with 10 mL lignin amine.

To evaluate their optical properties, the photoluminescence (PL) spectra of the obtained ZnO were measured at room temperature using a He-Cd laser as the excitation source at 325 nm. Figure 7a illustrates the PL spectra of ZnO-5, ZnO-10, and ZnO-15. A strong and sharp emission at about 389 nm was observed for all these ZnO, as well as a relative weak and broad green emission centered at about 550 nm. We have attributed the 389-nm UV emission to the direct recombination of the conduction band electrons and the valence band holes. This eigen-emission energy (389 nm/3.19 eV) is comparable to the band gap energy of 3.37 eV of typical ZnO materials. The weak and broad emission at about 550 nm is a visible emission, which originates from the transition of an excited electron from the conduction band of the nanomaterials to their defects having relatively higher energy levels than the valence band [19,20]. It is obvious that ZnO-5 shows lower intensities of both UV emission and visible emission, which suggests that the recombination of photogenerated charge carriers was inhibited [21]. In contrast, ZnO-10 and ZnO-15 show much more enhanced visible emission. This implies that the use of more than 10 mL lignin amine can make more defects on the ZnO crystallite surface.

We also determined the PL spectra of the ZnO prepared with 10 mL lignin amine at various molar ratios of Zn^2+^/OH^-^ as the ratio affects the defects of the synthesized materials. From Figure 7b, we can see that the basic concentration has a more pronounced effect on the intensity of the visible emission than on that of the UV emission. Upon increasing the Zn^2+^/OH^-^ from 1:2, 1:6, to 1:10, the intensity of the visible emission is generally quenched, accompanied with slight variation of UV emission. It is worth noting that the ZnO prepared at 1:4 displays the highest visible emission, as seen in Figure 7b. Associated with the above assignment of emissions, it is suggested that the ZnO prepared at 1:4 of Zn^2+^/OH^-^ tends to have more surface defects.

## Conclusions

With the aid of lignin amine, the flower-like ZnO nanomaterials featured with tapered petals have been synthesized by a facile precipitation method. The results of combined XRD and SEM have shown that the non-calcined and calcined ZnO nanomaterials have quite similar crystallinity, morphology, and particle size. This confirms that the non-calcined ZnO nanocrystallites are of high purity and well crystallized. Thus, applying our synthetic approach, the high-temperature calcining purification procedure that is usually used in many syntheses is no longer required. Furthermore, our approach greatly simplifies the synthesis of ZnO nanomaterials.

It has been revealed from the SEM images that the morphology and size of the synthesized ZnO crystallites can be tuned by the added lignin amine. We also found that both the lignin amine amount and molar ratio of Zn^2+^/OH^-^ have a significant effect on the PL spectra of ZnO, especially for the visible emission. The ZnO prepared with 10 mL lignin amine and Zn^2+^/OH^-^ of 1:4 has the favorable flower-like morphology with the proper crystalline size and also has the most defects, for it displays the most intensive visible emission band.

In brief, the lignin amine used in the present synthesis was obtained by chemically modifying abundant and cost-effective lignin. Therefore, the present study not only provides the possibility of large-scale production of ZnO nanoparticles but renews pulp industrial lignin waste and reduces environmental contamination as well.

## Competing interests

The authors declare that they have no competing interests.

## Authors’ contributions

TTM carried out the synthesis experiments and drafted the manuscript. DXS and CL carried out the degradation experiments. YLM and GZF participated in the sequence alignment. YRG participated in the design of the study and performed the analysis. QJP conceived the study and participated in its design. All authors read and approved the final manuscript.
